# Ordered Domains and Microwave Properties of Sub-micron Structured Ba(Zn_1/3_Ta_2/3_)O_3_ Ceramics Obtained by Spark Plasma Sintering

**DOI:** 10.3390/ma12040638

**Published:** 2019-02-20

**Authors:** Fei Liu, Shaojun Liu, Xuejiao Cui, Lijin Cheng, Hao Li, Jie Wang, Weidong Rao

**Affiliations:** 1 Shenzhen Research Institute, Central South University, Shenzhen 518057, China; 163311042@csu.edu.cn; 2 State Key Laboratory for Powder Metallurgy, Central South University, Changsha 410083, China; cxjsydl515@csu.edu.cn (X.C.); lihao92@csu.edu.cn (H.L.); zlyc135@163.com (J.W.), 183311055@csu.edu.cn (W.R.); 3 State Key Laboratory of Materials Processing and Die & Mould Technology, School of Materials Science and Engineering, Huazhong University of Science and Technology, Wuhan 430074, China

**Keywords:** B-site ordering, microwave ceramics, spark plasma sintering, domains, sub-micron sized grains, quality factor

## Abstract

The degree of Zn^2+^ and Ta^5+^ ions ordering could play an important role in the dielectric loss in Ba(Zn_1/3_Ta_2/3_)O_3_ (BZT) ceramics. However, the influence of the grain size of Ba(B′_1/3_B″_2/3_)O_3_ ceramics with nano or sub-micron grains on the ordering domains structure is still not clear. In the present paper, highly dense (~98%) BZT microwave dielectric ceramics with homogeneous sub-micron structure (~330 nm) were prepared through spark plasma sintering (SPS). High resolution transmission electron microscopy combined with X-ray diffraction (XRD)clearly showed that the B-site ordering structure of sintered BZT samples by SPS becomes the B-site long-range 1:2 ordering as annealing proceeds. In contrast, the short-range 1:2 ordering in non-annealed counterparts was also present, which was not detectable by XRD. The size of B-site ordering domains enlarged with annealing temperature. The sub-micron structure of sintered BZT ceramics by SPS remained stable at up to 1400 C; however, the size of B-site 1:2 ordering domain was more than five times larger, which led to a significant increase of the quality factor (*Q·f*) to 37,700 GHz from 15,000 GHz.

## 1. Introduction

Complex perovskite Ba(B′_1/3_B″_2/3_)O_3_ ceramics have become one of the most important microwave dielectric ceramics for wireless communication systems due to their very low dielectric loss or high quality factor (*Q**·**f*), relatively high dielectric constant (*ε_r_*), and near-zero temperature coefficient of resonant frequency (*τ_f_*) [1,2,3]. Ba(Zn_1/3_Ta_2/3_)O_3_ (BZT) microwave ceramics, as dielectric resonators and filters, have been broadly utilized in the wireless technologies [4]. It has been accepted that the quality factor of BZT ceramics is relevant to the degree of B-site {…Zn^2+^--Ta^5+^--Ta^5+^…} 1:2 ordering [5,6,7], ordering domain size, grain size and crystal defects [8,9,10,11,12], which are highly dependent on the sintering and annealing conditions. An initial investigation showed that in order to enhance the B-site 1:2 ordering degree, high annealing temperature (~1450 °C) and long soaking time (~48 h) are necessary [13,14,15]. Tamura et al. further showed that BaZrO_3_ doping significantly shortens necessary annealing time to obtain high *Q**·**f* values [16]. Furthermore, Davies et al. observed that Zr^4+^ ion could wreck the B-site 1:2 ordering [8]. With the increasing doping amount of Zr^4+^ ion, BZT ceramics gradually change from B-site 1:2 ordering to B-site 1:1 ordering. They attributed the improved *Q* value to the segregation of Zr^4+^ ion to the boundary region to stabilize the domain boundaries.

Although the complex relationship between quality factor and ordering domain is to be expected, this progress is impeded by extrinsic losses including the secondary phase, porosity, impurities, oxygen vacancies, and dislocations [17]. Additionally, dielectric loss in Ba(B′_1/3_B″_2/3_)O_3_ perovskite ceramics is significantly affected by ordering domain boundaries and grain boundaries [8]. However, there is a controversy in the effect of grain boundaries of microwave ceramics on their quality factor compared with that of ordering domain boundaries. We would like to mention that the number of grain boundaries has long been suspected to increase dielectric loss. Chen et al. suggested that improved *Q* values result from the grain growth that accompany a decrease in the amount and density of grain boundaries due to elevated annealing temperature and lengthy annealing time [18]. Similarly, Prasad et al. reported that the quality factor of BiVO_5_ ceramics increases with grain growing [19]. On the contrary, Kim et al. found that the quality factor of Ba(Ni_1/3_Nb_2/3_)O_3_ samples decreased with increasing grain size [20]. Due to the strict sintering condition (1520 °C for 48 h), BZT ceramic synthesized via traditional solid-state reaction (SSR) has a relatively large grain size (~5 µm). After heat-treatment, the grain size of highly ordered BZT would slightly and continuously grow (>5 µm) [21]. As a consequence, it is hard to determine the influence of grain boundaries on the quality factor [22,23,24]. 

Recently, spark plasma sintering (SPS) technique, as one of the field assisted sintering techniques, has received special attention because it can be used to effectively tailor microstructure at a relatively low temperature for a significantly decreased soaking time [25,26,27,28]. Cheng et al. found that sub-micron structured 0.7CaTiO_3_-0.3NdAlO_3_ microwave ceramics can be fabricated by SPS technology [26]. Undoped BZT microwave dielectric ceramics of >98% relative density with homogeneous sub-micron size grain (<300 nm) have been successfully prepared by spark plasma sintering [29]. Preliminary investigation by Raman spectra suggested that B-site 1:2 ordering is present in sintered ceramics by SPS. Additionally, the quality factor values of sintered BZT ceramics by SPS increase with annealing time. As expected, it is further improved to ~39,865 GHz by post-annealing at 1200 °C for 20 h in oxygen. It is speculated that the improved *Q·f* values of sintered BZT ceramics are closely related to the increasing ordering domain size during the annealing processing; however, a crucial issue is that the evolution of B-site 1:2 ordering degree and ordering domains needs to be clarified in this processing for polycrystalline Ba(B′_1/3_B″_2/3_)O_3_ ceramics with grain size falling to sub-micron level. Considering the high grain boundaries density in sub-micron structured BZT ceramics, another question that should be solved is what is the effect of grain-boundaries on the quality factor in Ba(B′_1/3_B″_2/3_)O_3_ ceramics with sub-micron grain size.

Using BZT ceramics with sub-micron structure as a prototype of Ba(B′_1/3_B″_2/3_)O_3_ ceramics, the present paper aimed to investigate the evolution of B-site ordering and ordering domains in sub-micron structured BZT ceramics fabricated by SPS during the annealing processing. Special attention was paid to the relationship between the ordering domain, grain size and dielectric loss of sub-micron structured BZT perovskite microwave dielectrics.

## 2. Experimental 

**Figure 1:**
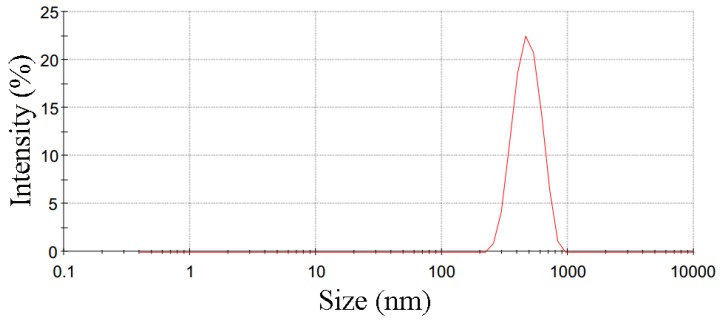
The size and size distribution of calcined powder.

In order to synthesize the nano-sized BZT powders, they were prepared by traditional route with mechanical milling processing. BaCO_3_, Ta_2_O_5_, and ZnO (99.99%) powders were used as raw materials. They were ball milled in ethanol at a speed of 300 rpm for 24 h. Then, the dry powders were re-milled at a speed of 500 rpm for 12 h. Then, the powder was calcined at 1350 °C for 5 h to synthesize BZT powder. Subsequently, the calcined powder was milled again with ethanol at a speed of 300 rpm for 24 h to optimize the size of powder and dried in air. Figure 1 shows the size and size distribution of synthesized BZT powder. The average size of as-synthesized is about 482 nm.

There is a densification stage and a non-densification stage (grain growth and pores elimination) in the sintering processing of SPS [25]. Our previous investigation showed that it is preferable to prepare BZT ceramics close to the temperature consistent with maximum densification rate in order to attain nano and/or sub-micron structure BZT ceramics without significant growth [29]. As a result, in order to reduce the volatilization of ZnO and obtain dense sub-micron structured BZT ceramics, 1300 °C was selected as the appropriate sintering temperature in the present investigation. As-refined BZT powder was sintered by SPS (FCT Systeme GmbH, Frederick, Germany) at 1300 °C for 10 min with a rate of 100 °C/min under a pressure of 30 MPa. The material of mold is graphite. All the samples sintered by SPS in vacuum. The diameter of the sintered samples by SPS was 20 cm; the thickness of measured samples was 5 cm. The sintered BZT samples were subsequently annealed at different temperature (from 1200 to 1400 °C) for 12 h in air. Additionally, before each measurement, all the samples by SPS were re-oxidized in air at 850 °C for 5 h.

The relative density of the sintered BZT samples was calculated by the Archimedes’ theory. The X-ray diffraction XRD technique (XRD, Rigaku Ultima IV, Tokyo, Japan) was used to examine the structure and B-site 1:2 ordering. Microstructure of thermally sintered and annealed BZT ceramics was surveyed by Scanning Electron Microscope (SEM, Model Quanta Quanta 250 FEG, FEI, Hillsboro, OR, USA). Ordering domain structure of annealed BZT ceramics was characterized by the spherical aberration corrected Transmission Electron Microscopy (TEM, Titan G2 60-300, FEI) with correction value (C3) of −500 nm.

The dielectric constant (*ε_r_*) and quality factor (*Q**·**f*) of the samples were measured by a network analyzer (N5230A, Agilent Santa Clara, CA, USA) using the Hakkicoleman dielectric resonator method modified by Courtney [30,31].

## 3. Results and Discussion

**Figure 2:**
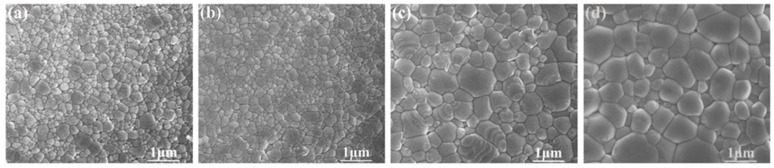
The microstructure of Ba(Zn_1/3_Ta_2/3_)O_3_ (BZT) ceramics by spark plasma sintering at different temperature for 10 min with 30 MPa applied pressure: (**a**) 1300 °C; (**b**) 1350 °C; (**c**) 1400 °C; (**d**) 1450 °C.

Figure 2 reveals the microstructure of sintered BZT samples by SPS (from 1300 to 1450 °C) under 30 MPa applied pressure for 10 min. Generally, the grain size of BZT samples is closely related to sintering conditions (temperature, time, pressure) during the SPS processing. A kinetic window exists for SPS, which can provide a chance to optimize microstructures of BZT samples with sub-micron grains [26]. As expected, the grain sizes of BZT samples rely on the sintering temperature. Abnormal grain growth of sintered BZT samples becomes obvious at 1400 and 1450 °C. Overall, fine and homogeneous grain size with sub-micron size (~340 nm) can be well maintained and kept stable for BZT ceramics sintered at 1300 and 1350 °C by optimizing the sintering parameters. It is noted that the sintering densities of sintered BZT ceramics present high sintering density up to 98%. 

**Figure 3:**
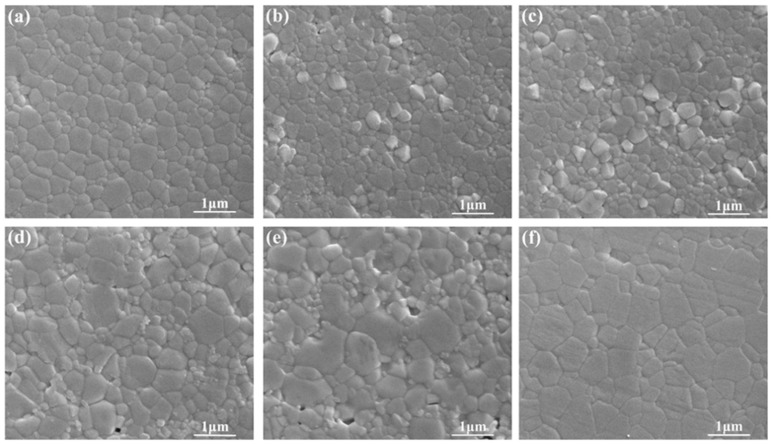
The microstructure of sintered BZT ceramics by spark plasma sintering (SPS) and annealed BZT ceramics at different temperature: (**a**) the sintered sample without annealing; (**b**) 1200 °C-12 h; (**c**) 1250 °C-12 h; (**d**) 1300 °C-12 h; (**e**) 1350 °C-12 h; (**f**) 1400 °C-12 h.

**Figure 4:**
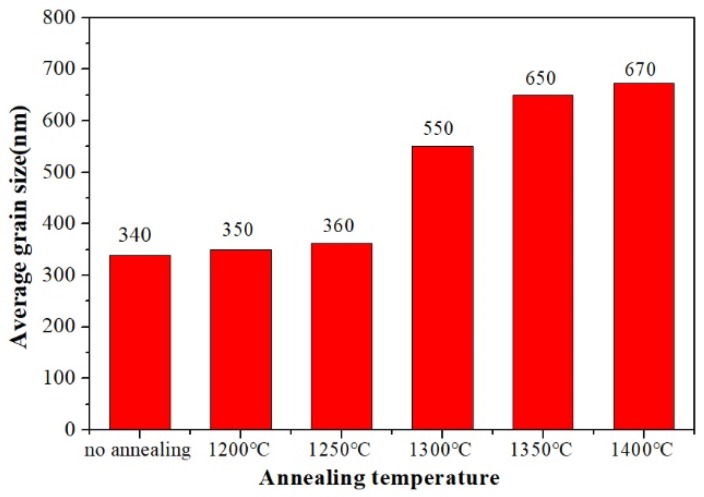
The grain size of sintered BZT ceramics by SPS at 1300 °C and annealed BZT ceramics at different temperatures.

One of the goals of this work was to investigate the evolution of the ordering domain during the annealing processing in sub-micron structured sintered BZT samples. The evolvement of microstructure of sintered BZT samples in a range of annealing temperature from 1200 to 1400 °C is illustrated in Figure 3. Although all the samples presented dense microstructures without abnormal grain growth compared to the unannealed one, slight grains growth in BZT ceramics annealed at 1200 and 1250 °C was observed. Furthermore, grains grow in BZT ceramics annealed at 1300 °C. The average grain sizes of sintered and annealed BZT ceramics are further summarized in Figure 4. As shown, there was a very slight increase in grain size of BZT ceramics annealed below 1250 °C, from 340 to 360 nm. In contrast, the grain growth was apparently accelerated for BZT ceramics annealed above 1300 °C. The average grain size increased from ~360 to ~550 nm when the annealing temperature increases to 1300 from 1250 °C. It further increased to 650 nm for BZT ceramics annealed at 1350 °C. This clearly indicates that there as a very narrow temperature window in which the grain growth was not sensitive to the annealing temperature of sintered BZT samples with sub-micron structure.

**Figure 5:**
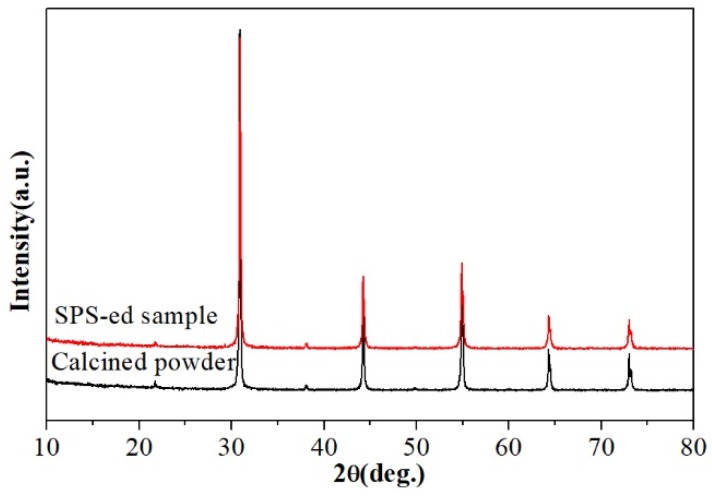
X-ray powder diffraction patterns of the calcined BZT powder and the sintered BZT ceramics by SPS.

Figure 5 shows the XRD patterns of BZT powder and BZT ceramics sintered at 1300 °C with 30 MPa applied pressure for 15 min. It is well known that main characteristics of the B-site cations ordering in XRD pattering include the appearance of the superlattice peaks resulting from the B-site 1:2 ordering [8]. As shown in Figure 5, no characteristic feature of the B-site 1:2 ordering structure was found. However, it is well known that XRD is broadly utilized to survey B-site ordering in Ba(B′_1/3_B″_2/3_)O_3_ perovskite ceramics; however, its accuracy is usually restricted by scattering length and volume fraction [14]. Our previous investigation showed that there exists B-site 1:2 ordering in sintered BZT ceramics that can be detectable by Raman spectroscopy [29]. The B-site 1:2 ordering occurs on a length scale that is so small that it cannot be found by XRD. The existence of short-range B-site 1:2 ordering was further confirmed by electron diffraction patterns collected along the [110] zone axis for the sintered BZT ceramics. It is noted that there are no obvious secondary phases that could play an important role in remarkably increasing the dielectric loss of BZT ceramics in the detectable range [32]. This fact excluded the possible influence of secondary phases or non-stoichiometry on the B-site 1:2 ordering and microwave dielectric properties.

**Figure 6:**
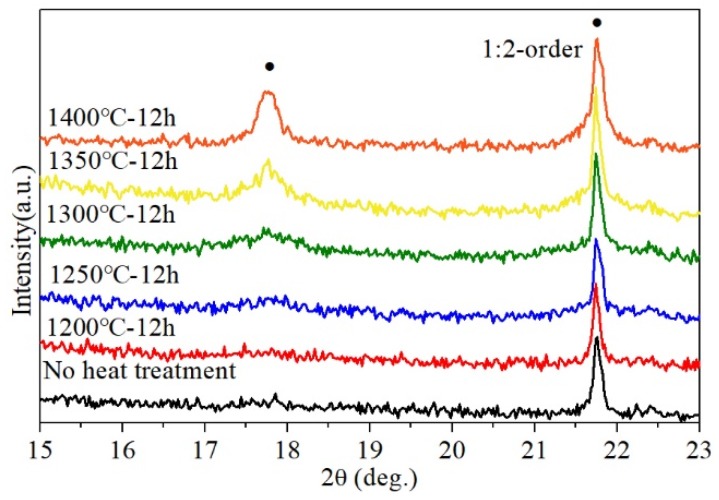
X-ray powder diffraction patterns (2θ=15°–23°) of sintered BZT ceramics by SPS at 1300 °C and annealed BZT ceramics at different temperatures. Superlattice reflections from 1:2 order are highlighted.

It is well-known that the ordering domains can be induced by proper annealing treatment. The B-site 1:2 ordering degree of BZT samples depends on the subsequent annealing treatment as well. The XRD patterns of the sintered BZT ceramics that are subsequently annealed at different temperature for 12 h are shown in Figure 6 (2θ = 15° to 23°). In this figure, XRD patterns of the sintered sample without annealing are also shown for comparison. As shown in Figure 6, obvious superlattice peaks stemming from the B-site 1:2 ordering can be found in the sintered BZT ceramics that were annealed at temperature > 1250 °C. This indicates that the crystal structure of BZT ceramics annealed in a range of 1300 to 1400 °C can be identified as the ordered type (P$\bar{3}$m1). The change of these microstructures is also surveyed by the diversity in the width and height of the superlattice reflections peaks [24]. In addition to line narrowing associated with domain sizes increasing, it was noted that the intensity of the superlattice peaks became stronger with increasing annealing temperature. These imply the nucleation and growth of ordering domains, which were dominated by kinetic rather than thermodynamic factors and dependent on annealing time and temperature. In contrast, no superlattice peaks were observed in the sintered BZT ceramics annealed blow 1250 °C. Thus, the existence of B-site 1:2 ordering in the BZT ceramics above was further confirmed by electron diffraction patterns.

**Figure 7:**
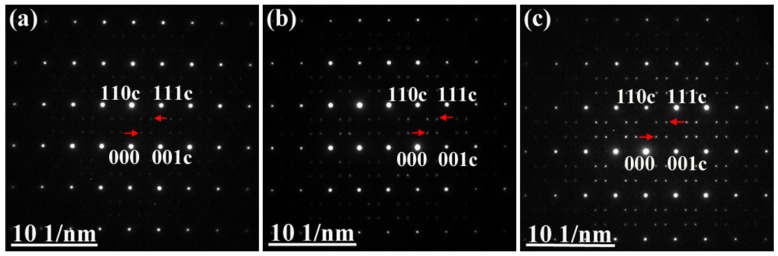
The different intensities of electron diffraction patterns collected along the [110] zone axis for the ordered BZT ceramics: (**a**) the sintered sample without annealing; (**b**) the annealed sample at 1300 °C for 12 h; (**c**) the annealed sample at 1400 °C for 12 h.

TEM provides a stronger tool to reveal the evolution of B-site 1:2 ordered domain and domain boundaries in BZT ceramics. TEM is especially well-suited to survey the B-site ordering of complex perovskites [33,34]. Selected area electron diffraction (SAED) patterns of BZT ceramics observed along the {110} crystal zone axes are shown in Figure 7, which are indexed by reference to the reciprocal lattice of cubic perovskite structure. The very weak superlattice reflections of the unannealed BZT ceramics, described in Figure 7a, were observed for the cubic perovskite structure. The red arrows show the diffraction spots of the superlattice. It clearly indicates that B-site 1:2 ordering in BZT ceramics fabricated by SPS at 1300 °C for 15 min with 30 MPa can be achieved. This is consistent with our previous Raman spectroscopy observations [29]. It also implies that the size of the ordering domain is so small that cannot be detected by XRD. As illustrated in Figure 7b, BZT ceramics annealed at 1300 °C has stronger superlattice reflections than unannealed ones. As shown in Figure 7c, we also noticed that the superlattice reflections became stronger with increasing annealing temperature.

**Figure 8:**
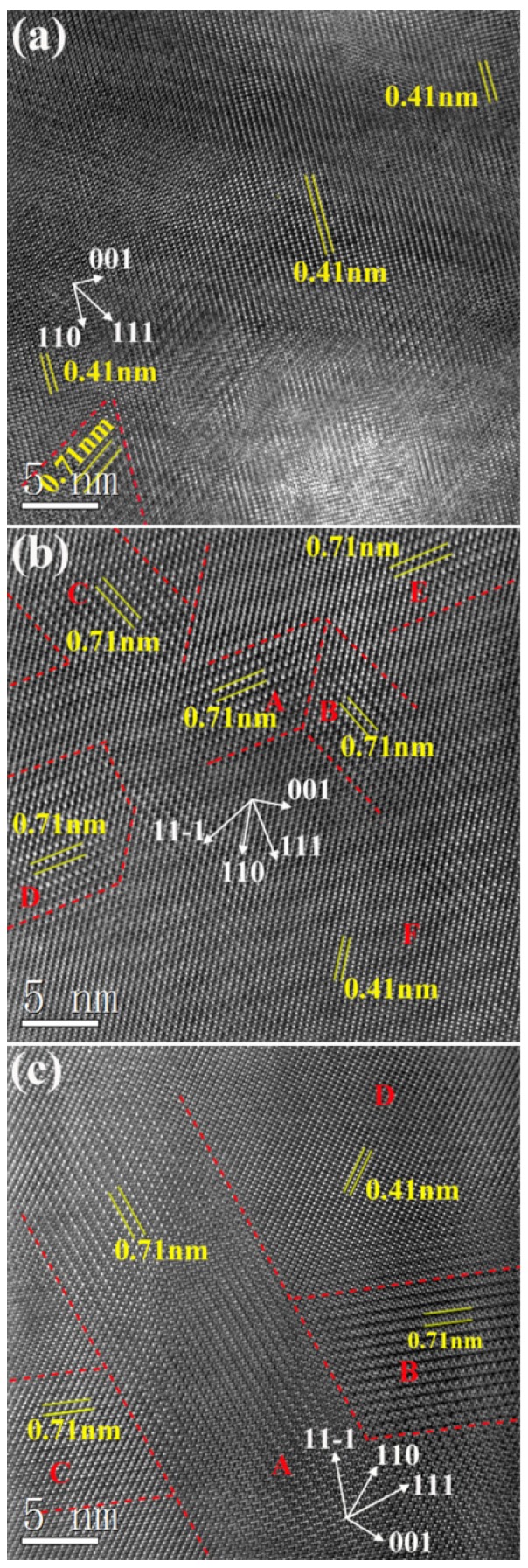
Lattice image viewed along the [110] zone axis for the ordered BZT ceramics: (**a**) the sintered sample without annealing; (**b**) the annealed sample at 1300 °C for 12 h; (**c**) the annealed sample at 1400 °C for 12 h.

High resolution transmission electron microscopy (HRTEM) was further utilized to characterize the domain of 1:2 Zn^2+^ and Ta^5+^ ions ordering of BZT samples [35,36]. The lattice images of ordered BZT ceramics, which were observed by HRTEM along the [110] zone axis, are shown in Figure 8. Although very weak superlattice diffraction was found in some areas of the sintered ceramics without annealing, ordering domains structure can be clearly observed in Figure 8a. The small size domain in the sintered BZT ceramics could be observed in the lower left corner in Figure 8a, where contrast-modulated structures along the [111] direction were observed. The lattice fringes along both [110] and [001] directions were discernible as well. However, the length of the ordering domains was very small. The interplanar distance of the contrast-modulated structures was found to be ~0.71 nm for the 1/3 [111] superlattice reflection though measuring the corresponding diffraction pattern. The other lattice image in Figure 8a was classified as disordering structure whose interplanar spacing was ~0.41 nm. 

In contrast, the density of ordering domains in the annealed BZT ceramics at 1300 °C significantly increased, although the size and shape of ordering domains were slightly irregular, as shown in Figure 8b where they are marked by depicting domain boundaries with red dotted line to separate the various orientation variants. The diversities in areas A and B derived from the B-site ordering along each of the two in-plane [111] directions of the cubic structure, in addition to three ordered domains labeled C, D, E in Figure 8b. It should be pointed out that there were also some domain boundaries that have not been marked, since the boundaries between the domains of ordering and the areas of disordering cannot be identified finitely. The average length of the ordering domains was ~8 nm. Compared with sintered BZT ceramics without annealing, the size of domains of the annealed ones at 1300 °C was slightly larger. We also found that the regions of ordering domains in Figure 8b possesses about 50% of the area of this image, which was much larger than that of the sintered BZT ceramics without annealing. It is clear that annealing at 1300 °C was more beneficial to the formation of ordering domains, rather than the growth. We would like to mention the type of B-site ordering domain structure of sintered BZT samples with sub-micron structure was the same as that prepared by conventional solid-state reaction method [10].

As the temperature of annealing rose to 1400 °C, the size of the ordering domains labeled A, B, C of annealing samples continued to grow, and the domains possessed about 75% of the area of the image, as shown in Figure 8c. In addition, the shape of domains became more regular and was close to a rectangle. There are two possible types of domain boundaries in BZT perovskite ceramics, including twin boundary and anti-phase boundary [8]. Unlike the type of ordering domains of annealing samples at 1300 °C, anti-phase boundary marked by red dotted line is dominant in this image. The average size of domains was found to be >40 nm and the domains of this sample were much larger than that of the sample annealed in 1300 °C.

With the growth of ordering domains, the number of domain boundaries gradually decreased. The reduction of the number of domain boundaries was be benefit to the improvement of the quality factor. It is well known that there are very high losses at the domain boundary regions due to the elastic strain, unfavorable coulombic interaction and chemical inhomogeneity in Ba(B′_1/3_B″_2/3_)O_3_ perovskite ceramics [8]

The grain size and ordering domain size derived from SEM and TEM observations for the sintered BZT ceramics with and without annealing are summarized in Table 1. *Q**·**f* value of BZT ceramics annealed at 1300 °C slightly increased to 23,600 from 15,000 GHz, compared to that of the sintered sample. Their average grain sizes increased to ~550 nm, though still within the submicron range, and there occurred a number of small domains with an average size of ~8 nm. When the annealing temperature rose to 1400 from 1300 °C, *Q·f* value of sintered BZT ceramics grew to 37,700 from 23,600 GHz. The size of B-site ordering domain was augmented from 8 to 40 nm and the grain size creot to 673 from 551 nm. It is worth noting that the relative growth degree of the domain size was much bigger than that of the grain size. It coincided with the XRD results shown in Figure 6 that the intensity of superlattice peaks tended to be stronger as annealing temperature rose, indicating that the domain size of samples became larger. In addition, the values of the dielectric constant and temperature coefficient of all samples were relative stable in submicron structured BZT ceramics. Annealing treatment had no effect on dielectric constant and temperature coefficient.

Therefore, it can be inferred that, within submicron scale, dielectric loss is slightly reduced at low annealing temperatures due to the transformation from disordering to local B-site ordering and increase in number of domains. With annealing temperature rising, the domains significantly grow and the density of boundaries reduces, so as to effectively improve the *Q* value. It is believed that the size of domain structure possibly plays a leading role in the reduction of dielectric loss in submicron structured BZT ceramics.

## 4. Conclusions

Highly dense (~98%) BZT microwave dielectric ceramics with homogeneous sub-micron sized grains (~330 nm) and B-site 1:2 ordering were prepared by SPS. The size of B-site 1:2 ordering domains in sintered Ba(Zn_1/3_Ta_2/3_)O_3_ ceramics by SPS following annealing processing increased. In contrast, the B-site 1:2 ordering structure comprising small ordering domains in non-annealed counterparts was present that was not detectable by XRD. It was observed that the grain size of sub-micron structured Ba(Zn_1/3_Ta_2/3_)O_3_ by SPS remained stable up to 1400°C; however, the size of B-site ordering domain was more than five times larger (from ~8 to >40 nm), which led to a significantly increase of the quality factor (*Q**·**f*) to 37,700 from 23,600 GHz. Our results show that the ordering induced domains rather than grain boundary might play a leading role in increasing the quality factor of BZT microwave ceramics in the submicron range.

## Figures and Tables

**Table 1:** Summary of high resolution transmission electron (HRTEM) results and microwave dielectric properties of Ba(Zn_1/3_Ta_2/3_)O_3_ prepared by spark plasma sintering and annealed at different temperature for 12 h.

Anneal Temperature (°C) and Time (12 h)	Grain Size (nm)	Domain Size (nm)	*Q·f* (GHz)
/	340	~5	15,000
1200 °C	350	/	18,300
1250 °C	360	/	21,000
1300 °C	550	~8	23,600
1350 °C	650	/	30,100
1400 °C	670	>40	37,700
